# Global prevalence of falls in the older adults: a comprehensive systematic review and meta-analysis

**DOI:** 10.1186/s13018-022-03222-1

**Published:** 2022-06-28

**Authors:** Nader Salari, Niloofar Darvishi, Melika Ahmadipanah, Shamarina Shohaimi, Masoud Mohammadi

**Affiliations:** 1 Department of Biostatistics, School of Health, Kermanshah University of Medical Sciences, Kermanshah, Iran; 2 Student Research Committee, Kermanshah University of Medical Sciences, Kermanshah, Iran; 3 Student Research Committee, Shahid Beheshti University of Medical Sciences, Tehran, Iran; 4 Department of Biology, Faculty of Science, University Putra Malaysia, Serdang, Selangor Malaysia; 5 Cellular and Molecular Research Center, Gerash University of Medical Sciences, Gerash, Iran

**Keywords:** Fall, Prevalence, Accident, Systematic review, Meta-analysis

## Abstract

**Background:**

With increasing life expectancy, declining mortality, and birth rates, the world's geriatric population is increasing. Falls in the older people are one of the most common and serious problems. Injuries from falls can be fatal or non-fatal and physical or psychological, leading to a reduction in the ability to perform activities of daily living. The aim of this study was to determine the prevalence of falls in the older people through systematic review and meta-analysis.

**Methods:**

In this systematic review and meta-analysis, the data from studies on the prevalence of falls in the older people in the world were extracted in the databases of Scopus, Web of Science (WoS), PubMed and Science Direct, and Google Scholar, Magiran and Scientific Information Database (SID) without any time limit until August 2020. To analyze the eligible studies, the stochastic effects model was used, and the heterogeneity of the studies with the I^2^ index was investigated. Data analysis was conducted with Comprehensive Meta-Analysis software (Version 2).

**Results:**

In the review of 104 studies with a total sample size of 36,740,590, the prevalence of falls in the older people of the world was 26.5% (95% CI 23.4–29.8%). The highest rate of prevalence of falls in the older people was related to Oceania with 34.4% (95% CI 29.2–40%) and America with 27.9% (95% CI 22.4–34.2%). The results of meta-regression indicated a decreasing trend in the prevalence of falls in the older people of the world by increasing the sample size and increasing the research year (*P* < 0.05).

**Conclusion:**

The problem of falls, as a common problem with harmful consequences, needs to be seriously considered by policymakers and health care providers to make appropriate plans for preventive interventions to reduce the rate of falls in the older people.

## Background

Rising life expectancy and rising mortality are contradictory, and aging is a critical period in human life during which changes occur in internal and external organs. These changes cause the individual to adapt to the environment. Throughout the world, the world's geriatric population is rising as increasing life expectancy, declining mortality, and birth rates. Also, the number of people over the age of 60 is growing faster than other age groups. With this significant increase in the older people, improving their health and well-being is a priority [1]. According to studies, the geriatric population will increase from 600 million in 2000 to 1 billion and 200 million in 2025 [2].

One of the most common and serious problems among the older people is falling [1]. According to the World Health Organization (WHO), a fall is defined as an event that results in a person coming to rest inadvertently on the ground or floor or other lower level [3]. Injuries from falls can be fatal or non-fatal. Falls are associated with reduced quality of life and higher costs of health care. At older ages, the health effects and costs of falls are increasing significantly worldwide [4].

The fall can be due to factors such as medication, osteoarthritis, depression, dizziness, and disturbances in balance and gait (due to cerebellar damage or in connection with age-related degenerative changes in the middle and inner ear). Muscle weakness due to aging or medication can cause falls as well. The use of assistive devices, age over 80 years, postural hypotension and impaired vision (decreased adaptive power, lens opacity), and chronic diseases are among the causes of falls [5–7].

Injuries due to falls may lead to a decrease in the ability to perform activities of daily living [8]. Falls, especially in the older people, increase disability, and the injured people often do not recover to their previous functional level [9, 10]. In addition to physical injuries, falls also have psychological consequences [11]. In addition to physical injuries, falls also have psychological consequences [11]. Many people who have experienced a fall are afraid of falling, which in turn leads to immobility, followed by pressure ulcers, rhabdomyolysis, pneumonia, weakness, and increased risk of falls [12, 13]. Serious injuries caused by falls include fractures, especially pelvic and thigh fractures. Also, most injuries occur in the lower limbs, upper limbs, head, and trunk, which most of them are bruises or cuts, fractures, and dislocations [14, 15].

Among them 5% lead to fractures and 5–10% to other injuries. Among the causes of hospitalization, hospitalization due to fall is 5 times more than hospitalization due to other injuries [16]. The prevalence of falls in people over 65 is 30% in the USA, 13.7% in Japan, 26.4% in China, and 53% in India [17]. Research has also shown that the prevalence of falls is higher in older women than men [18].

The average fall in a nursing home is 1.5 falls per year per bed. Investigating and reducing risk factors reduces the risk of falls. Regular assessment in a nursing home can help identify high-risk patients [19]. The evaluation includes fall conditions, the patient's complete physical history, and search for possible risk factors. One of the most effective strategies for preventing falls is multi-factor interventions aimed at identifying risk factors, muscle strengthening exercises with balance training, and quitting psychedelic drugs [20, 21].

## Methods

### Searching strategy and study selection

The present study was conducted to investigate the prevalence of falls in the older people worldwide via systematic review and meta-analysis. To collect data in this study, international databases, Scopus, Web of Science, PubMed, Science Direct, Google Scholar, SID, Magiran were sought without any time limit until August 2020. The search process was carried out in the mentioned databases using the English keywords, "Prevalence;" "Fall"; "Slip"; "Older people"; "Older adult"; and the Persian keywords Fall; Accidents; Older people; and their possible combinations in international bases. For instance, how to search the PubMed database is described in the box below. To study the Gray literature, the review of related sites was also on the agenda. To maximize the comprehensiveness of the search, the list of the sources used in all related articles that were found in the above search was manually reviewed. Initially, the duplicate studies in various searched databases were excluded from this study. Then, the researchers of this study prepared a list of titles of all the remaining articles to obtain eligible articles by evaluating the articles in this list. In the first stage, screening, the title, and abstract of the remaining articles were carefully studied, and irrelevant articles were removed based on the inclusion and exclusion criteria. In the second stage, the evaluation of the suitability of the studies, the full text of the possible relevant articles remaining from the screening stage was examined based on the inclusion and exclusion criteria and in this stage, unrelated studies were eliminated. To avoid bias, all steps of reviewing sources and extracting data were performed by two researchers independently. In case any articles were not included, the reason for deleting them was mentioned. In cases where there was disagreement between the two researchers, the article was reviewed by a third party. A total of 104 studies entered the third stage, i.e., qualitative evaluation.

PubMed Search Strategy: (prevalence[Title] OR outbreak[Title]) AND (fall down[Title] OR slip[Title] OR fall[Title] OR damage[Title] OR accidental fall[Title] OR injury[Title] AND (older people[Title] OR older adult[Title] OR aged[Title]) OR (fall down[Title] AND older people[Title]) OR (slip[Title] AND older adult[Title]) OR (accidental fall[Title] AND aged[Title]).

### Inclusion and exclusion criteria

Inclusion criteria include: 1—cross-sectional studies, 2—studies that have studied the prevalence of falls in the older people worldwide, 3—observational studies (non-interventional studies), 4—Persian studies, 5—English studies, and exclusion criteria include: 1—case–control studies, 2—cohort, 3—case report, 4—interventional studies, 5—letter to editor, 6—studies whose full text is not available, 7—duplication of studies, 8—systematic review and meta-analysis studies.

### Qualitative evaluation

To validate and evaluate the quality of articles (i.e., methodological validity and results), a checklist appropriate to the type of study was used. The STROBE checklist is commonly used to critically and qualitatively evaluate observational studies such as the present study. The STROBE checklist consists of six general scales/sections: title, abstract, introduction, methods, results, and discussion. Some of these scales have subscales, and in total, this statement contains 32 items. In fact, these 32 items encompass various methodological aspects of the study, including title, problem statement, study objectives, type of study, the statistical population of the study, sampling method, determining the appropriate sample size, definition of variables and procedures, data collection tools, statistical analysis, and findings. Accordingly, the maximum score obtained from the qualitative assessment in the STROBE checklist will be 32. Considering the score of 16 as the cutoff point, those articles obtaining a score of 16 and above will be considered as articles with suitable and average methodological quality, and those obtaining below 16 were considered as poor and were therefore excluded from the study.

### Extracting the data

The information related to all selected articles which were entered into the systematic review and meta-analysis process was extracted from a pre-prepared checklist. This checklist includes the title of the article, the name of the first author, the year of publication, the country, the sample size, the number of falls per sample, the average age of the sample, and the prevalence and continent percentage.

### Statistical analysis

I^2^ test was used to evaluate the heterogeneity of selected studies. To investigate the dissemination error, due to the large statistical sample size included in the study, Begg and Mazumdar test was used at a significance level of 0.1 and its corresponding Funnel plot. The data were analyzed using the Comprehensive Meta-Analysis Software (Version 2).

## Results

### Study selection and data extraction

This study examined the prevalence of falls in the older people of the world through systematic review and meta-analysis. After searching in various databases, from a total of 4251 articles, 1795 articles from the PubMed database, 172 articles from the Science Direct database, 160 articles from the Scopus database, 160 articles from Web of Science database, and 1720 articles from Google Scholar database, 136 articles from Magiran database, and 111 articles from SID database were selected for the study. Out of a total of 4251 identified studies, 66 were duplicate and were excluded. In the screening stage, out of 4185 studies, 3651 articles were excluded through studying the title and abstract sections based on inclusion and exclusion criteria.

In the competency assessment stage, out of 540 studies, the remaining 436 articles were excluded regarding the inclusion and exclusion criteria due to being irrelevant through perusing the full text of the articles. In the qualitative evaluation stage, through studying the full text of the articles and based on the STROBE checklist, out of the remaining studies, no article was removed due to the poor methodological quality.

**Fig. 1:**
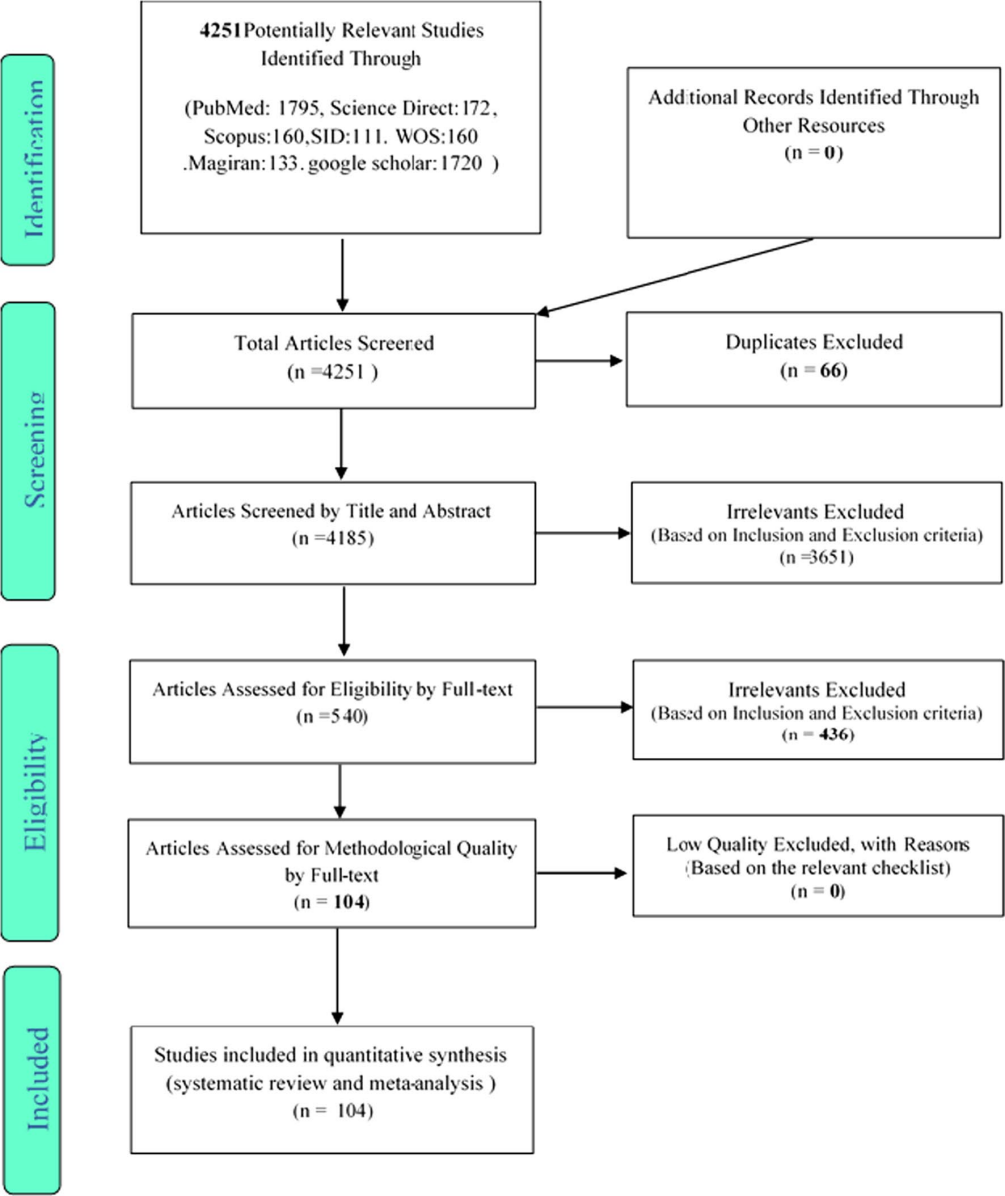
The flowchart on the stages of including the studies in the systematic review and meta-analysis (PRISMA 2009)

The studies were reviewed based on the four-step PRISMA 2009 process, including article identification, screening, review of article acceptance criteria, and finally, the articles entered to the meta-analysis (Fig. 1). Ultimately, 104 studies were included in the final analysis, the information of which was mentioned in the tables (Table 1) [14, 19, 22–123].

**Table 1:** The extracted data from the final studies entered into the meta-analysis

	Published in	First author	Country	Average age	Sample size	Number of falls	Prevalence	Continent
1	2012	Demura [14]	Japan	70.3 ± 6.8	1850	386	20.9	Asia
2	2016	Johansson [19]	Sweden	70	1350	148	11	Europe
3	2008	Steven [22]	USA	≥ 65	922,200	5.8 m	15.9	America
4	2004	Aktaş [23]	Turkey	78	32	8	25	Asia
5	2015	Al Tehewy [24]	Egypt	67.7	411	46	11.2	Europe
6	2018	Aljawadi [25]	Saudi	≥ 60	2964	388	13.2	Asia
7	2015	Almada [26]	Europe	70 ± 8.9	41,098	3452	8.4	Europe
8	2018	Almegbel [27]	Saudi Arabia	68.8 ± 9	1182	590	49.9	Asia
9	2019	Almeida [28]	Brazil	≥ 65	211	60	28.9	America
10	2013	Antes [29]	Brazil	70–7	1705	322	19	America
11	2004	Avdić [30]	USA	72.38 ± 5.9	77	21	27.77	America
12	2009	Barker [31]	Australia	81.59	87	46	52.87	Oceania
13	2010	Bauer 32]	Germany	75.6 ± 8.3	61	42	71.2	Europe
14	2010	Bekibele [33]	Nigeria	≥ 65	2096	482	23	Africa
15	1997	Berg [34]	USA	71.7	96	50	52	America
16	2004	Bergland [35]	Norway	80.8	307	155	50.8	Europe
17	2019	Bernard [36]	France	72.45 ± 5.1	1471	485	33	Europe
18	1988	Blake [37]	Colombia	≥ 65	1042	356	35	America
19	2009	Boyd [38]	USA	≥ 65	35 m	3.5 m	10	America
20	2009	Carpenter [39]	USA	≥ 65	263	102	39	America
21	2015	Cevizci [40]	Turkey	74.1 ± 6.8	1001	321	32.1	Asia
22	2011	Chin-Liang [41]	China	82.1 ± 5.1	371	33	8.9	Asia
23	2012	Da Cruz [42]	Brazil	69.7	420	135	32.1	America
24	2019	Del Brutto [43]	USA	70.4 ± 7.9	463	173	53	America
25	2011	Demura [44]	Japan	70.7 ± 7	968	150	15.49	Asia
26	2016	Dhargave [45]	India	74.61 ± 8.4	163	47	28.9	Asia
27	2019	Dias [46]	Brazil	73	211	60	28.9	America
28	2009	Divani [47]	New Zealand	74.4 ± 7.2	1104	408	37	Oceania
29	2019	Dos Santos [48]	Brazil	70	820	229	27.9	America
30	2018	Ehrlich [49]	USA	≥ 65	7601	1482	19.5	America
31	2018	Fahlström [50]	Sweden	≥ 65	148	117	79	Europe
32	2013	Fhon [51]	Brazil	73.5 ± 8.4	240	92	38.6	America
33	1996	Fletcher [52]	Canada	≥ 65	63	20	31.7	America
34	2016	Foran [53]	Ireland	≥ 65	753	200	26.7	Europe
35	2016	Gale [54]	England	≥ 50	4301	1144	28.4	Europe
36	2014	George [55]	USA	≥ 65	1653	294	18	America
37	2017	Handrigan [56]	Canada	≥ 65	15,860	3172	20	America
38	2013	Hanlin [57]	USA	73.2	103	55	54	America
39	2020	Henwood [58]	USA	62.5	237	134	57	America
40	2011	Holt [59]	New Zealand	≥ 65	101	35	35	Oceania
41	2013	Isenring [60]	Australia	74.3	254	73	28.6	Oceania
42	2019	Janakiraman [61]	Ethiopia	≥ 50	599	170	28.4	Africa
43	2002	Izumi [62]	Japan	75	746	93	12.5	Asia
44	2014	Kabeshova [63]	France	71 ± 5.1	1760	346	19.7	Europe
45	2011	Kadir [64]	Malaysia	67.5 ± 5.6	131	17	12.9	Asia
46	2015	Kamińska [65]	Poland	78.6 ± 7.4	304	233	76.6	Europe
47	2018	Kang [66]	China	67.4 ± 5.6	619	125	20.1	Asia
48	2012	Kantayaporn [67]	Thailand	75.35	10,329	1244	12.04	Asia
49	2020	Kim [68]	Korea	≥ 45	9279	347	3.7	Asia
50	2019	Kistler [69]	USA	54.5	181,208	47,894	26.4	America
51	2007	Laessoe [70]	Denmark	73.7	94	14	15	Europe
52	2018	Lastrucci [71]	Finland	77.8 ± 8.7	1220	142	11.6	Europe
53	2011	Lim [72]	Korea	73.5 ± 6.3	828	108	13	Asia
54	2020	Lin [73]	China	≥ 60	335	77	23.28	Asia
55	2012	Logiudice [74]	Australia	≥ 45	363	113	31	Oceania
56	2018	Mahmoodabad [75]	Iran	71.42 ± 5.9	200	60	30	Asia
57	2001	Milisemiller [76]	Canada	62 ± 15.7	435	228	52.4	America
58	2007	Milisen [77]	Belgium	67.2 ± 18.4	2568	136	5.29	Europe
59	2019	Ofori-Asenso [78]	USA	62	1019	445	43.7	America
60	2013	Orces [79]	Brazil	≥ 60	5227	1954	37.4	America
61	2018	Ouyang [80]	China	60.5 ± 9.2	12,527	2041	16.3	Asia
62	2014	Pal [81]	New Zealand	≥ 45	135	36	27	Oceania
63	2018	Pathania [82]	India	75.2	335	55	16.4	Asia
64	2017	Pereira [83]	Brazil	83.7	3496	164	46.9	America
65	2019	Pitchai [84]	India	69.6	2049	512	24.98	Asia
66	2004	Schoenfelder [85]	USA	84.1	81	42	53	America
67	2014	Schumacher [86]	Germany	65.7	862	30	3.5	Europe
68	2016	Secil [87]	Turkey	68.3 ± 3.2	343	124	36.2	Asia
69	2013	Seifer [88]	USA	77	81	21	25.9	America
70	2018	Sharif [89]	USA	≥ 60	370	188	50.8	Asia
71	2015	Sharifi [90]	Iran	76.2	194	52	27.3	Asia
72	2009	Shin [91]	Korea	72.82	335	48	15	Asia
73	2011	Siqueira [92]	Brazil	70.9	6616	1826	27.6	America
74	2012	Suzuki [93]	Japan	86.94	135	50	37.04	Asia
75	2018	Tanaka [94]	Japan	68.1	1561	437	28	Asia
76	1993	Topper [95]	USA	83	100	59	59	America
77	2014	Tsai [96]	China	≥ 65	775	378	48.8	Asia
78	2009	Vassallo [97]	UK	82.1	825	150	18.1	Europe
79	2018	Vieira [98]	Brazil	≥ 60	1451	407	28.1	America
80	2004	Weir 99]	Canada	≥ 65	73,113	62,146	85	America
81	2019	Whitney [100]	USA	≥ 65	7598	827	10.88	America
82	2016	Ylitalo [101]	USA	62	280,035	756	27	America
83	2009	Yu [102]	China	≥ 60	1512	272	18	Asia
84	2018	Zhou [103]	China	≥ 60	1557	227	17.8	Asia
85	2019	Bagheri Ruchi [104]	Iran	70.11	300	100	33.3	Asia
86	2014	Taheri Tanjani [105]	Iran	≥ 60	1323	337	25.5	Asia
87	2020	Habibeh [106]	Iran	67.04	400	110	27.5	Asia
88	2016	Hoseini [107]	Iran	69.37	1616	274	17	Asia
89	2016	Khazaee [108]	Iran	≥ 60	11,954	2581	21.59	Asia
90	2013	Jafarian amiri s.r. [109]	Iran	70.1	350	123	35.1	Asia
91	2007	Nader [110]	Iran	67	207	121	58.46	Asia
92	2017	Vakili Sadeghi [111]	Iran	≥ 60	1482	271	18.3	Asia
93	2018	Gorzin [112]	Iran	≥ 60	148	29	20.13	Asia
94	2015	Aghaee [113]	Iran	72.24	2336	1033	44.2	Asia
95	2016	Nabavi [114]	Iran	70.42	288	88	30.9	Asia
96	2015	Najafi Ghazalche [115]	Iran	67.63	160	15	9.4	Asia
97	2018	Naamani [116]	Iran	78 ± 8	400	112	28	Asia
98	2015	Borhani Nezhad [117]	Iran	78.65	204	69	33.8	Asia
99	2013	Iranfar [118]	Iran	≥ 60	400	292	73	Asia
100	2015	Ghodsi [119]	Iran	≥ 60	960	672	70	Asia
101	2015	Mazharizad [120]	Iran	≥ 60	300	141	47.3	Asia
102	2017	Hadinejad [121]	Iran	70 ± 9	77,576	24,824	32	Asia
103	2013	Safizadeh [122]	Iran	69.05 ± 7.9	11,120	1234	11.1	Asia
104	2015	Torkaman Gholami [123]	Iran	60–80	378	264	70	Asia

**Fig. 2:**
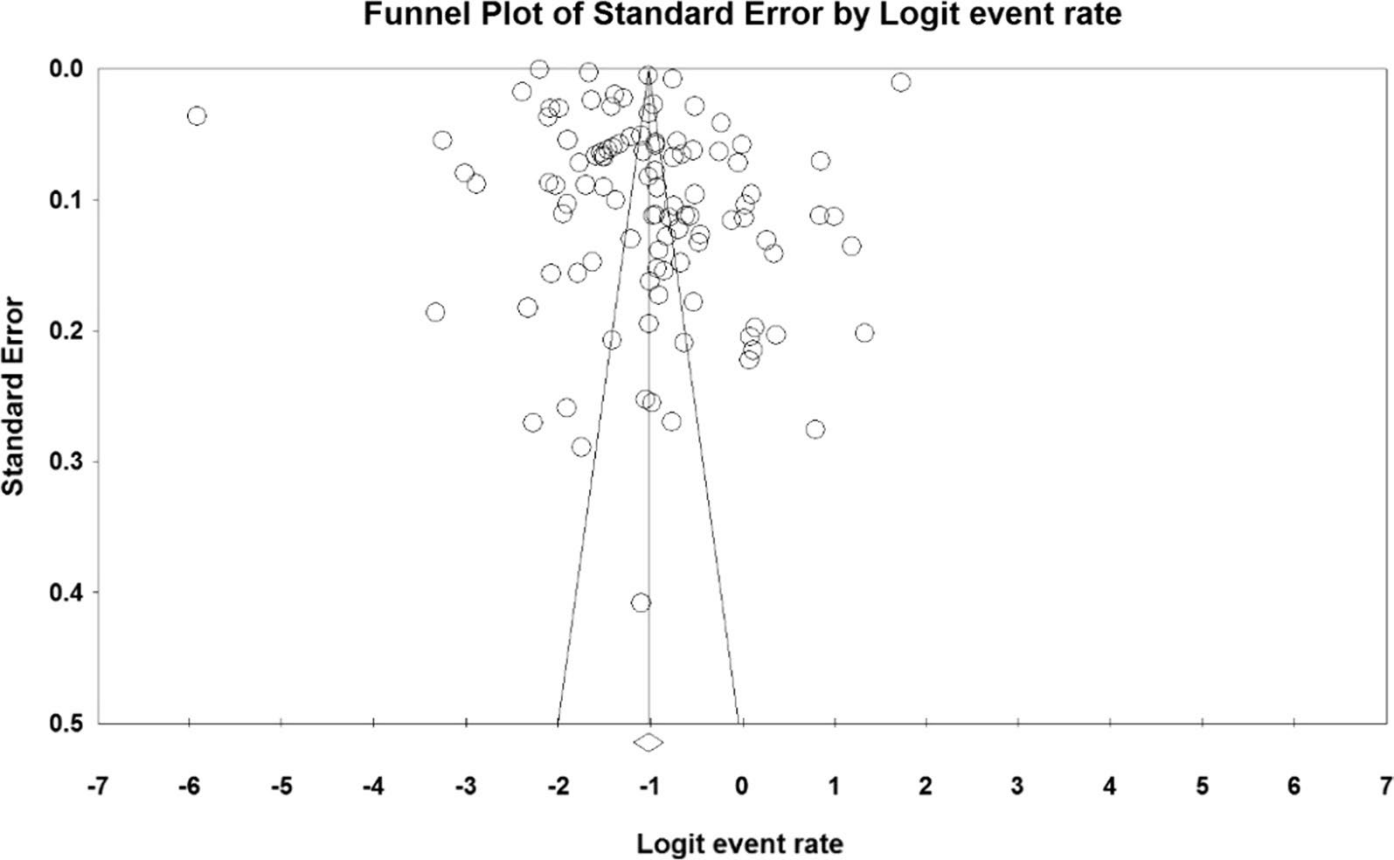
Funnel plot results of the prevalence of falls in the older people worldwide

The probability of bias in the dissemination of fall outcomes in the older people of the world by Funnel plot and Begg and Mazumdar test at a significance level of 0.1 indicated no dissemination bias in the present study (*P* = 0.101) (Fig. 2).

Based on the test results (I^2^: 99.9) and due to the heterogeneity of selected studies, a random-effects model was used to combine the studies and the shared prevalence estimate. The reason for heterogeneity between studies can be due to differences in sample size, sampling error, year of study, or place of study. Out of the 104 articles submitted for systematic review and meta-analysis with a sample size of 1,741,613 patients, 48 studies were conducted in Asia, 16 studies in Europe, 2 studies in Africa, 32 studies in America, and 6 studies in Oceania. The smallest and highest sample sizes were related to the studies of Aktaş, S. et al. (2004) (*n* = 32) [23] and J.A. Steven et al. (2008) (*n* = 922,200) [38]. The characteristics of the eligible studies shown in the meta-analysis are given in Table 1.

### Meta-analysis

**Fig. 3:**
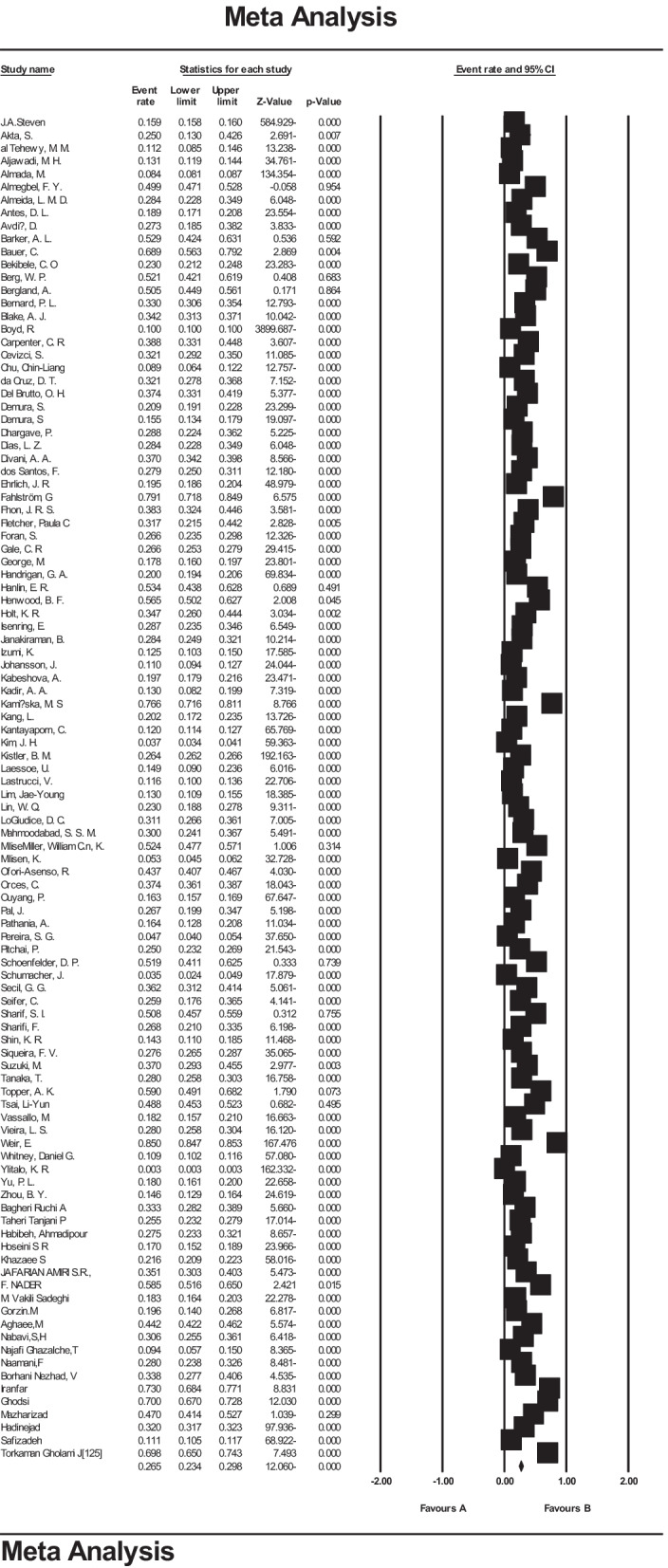
The prevalence of falls in the world's older people and 95% confidence interval based on a random-effects model

According to the results of the present study, the prevalence of falls in the world's older people was 26.5% (95% CI 23.4–29.8%). The midpoint of each line segment shows the prevalence in each study, and the diamond shows the population prevalence for the entire studies (Fig. 3).

### Meta-regression test

**Fig. 4:**
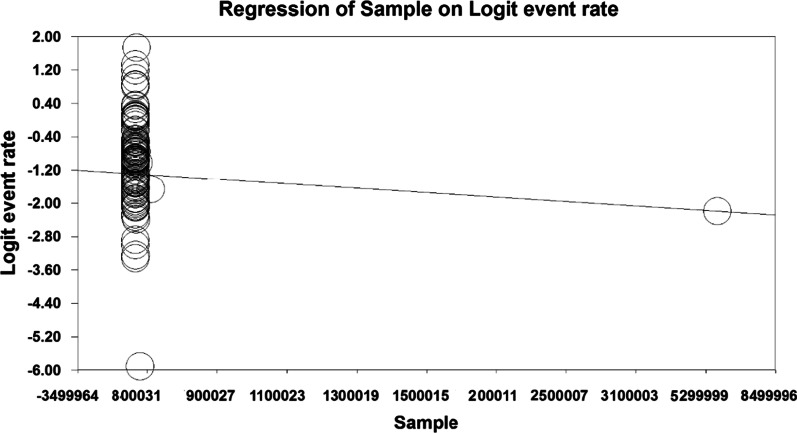
Meta-regression chart of the prevalence of falls in the older people of the world by sample size

**Fig. 5:**
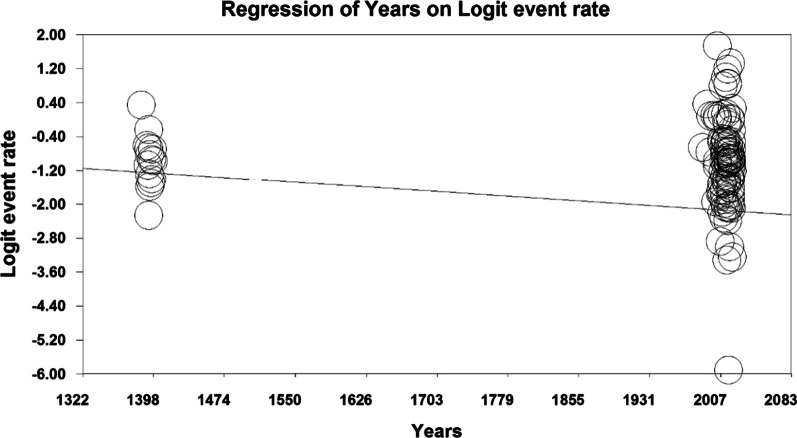
Meta-regression chart of the prevalence of falls in the older people of the world by the year

To investigate the effects of potential factors in the heterogeneity of the prevalence of falls in the older people in the world, meta-regression was used for the two factors of the sample size (Figs. 4, 5). According to Fig. 4, with increasing sample size, the prevalence of falls in the older people of the world decreases, which there is a statistically significant difference (*P* < 0.05). It was also reported (Fig. 5) that with the increase in the research year, the prevalence of falls in the older people of the world decreases, which there is also a statistically significant difference (*P* < 0.05).

### Subgroup Analysis

Table 2 reports the prevalence of falls in the world's older people in Asia, Europe, Africa, and America and Oceania. The highest rate of prevalence of falls in the older people was related to Oceania with 34.4 (95% CI 29.2–40) and America with 27.9 (95% CI 22.4–34.2) (Table 2). Table 2 is based on the studies performed, and in order to reduce the heterogeneity created in the whole study, as reported in Table 2, the number of studies does not have the same distribution and therefore the higher or lower prevalence in a continent. It is based only on studies of that continent.

**Table 2:** Prevalence of falls in the older people of the world according to different continents

Continents	Number of articles	Sample size	*I*^2^	Begg and Mazumdar test	Prevalence % (95% CI)
Asia	48	164,593	99.4	0.210	25.8 (95% CI 22.1–29.9)
America	32	36,513,725	99.9	0.109	27.9 (95% CI 22.4–34.2)
Europe	16	57,533	99.5	0.964	23.4 (95% CI 15.8–33.2)
Africa	2	2695	86.3	–	25.4 (95% CI 20.5–31)
Oceania	6	2044	79.4	0.573	34.4 (95% CI 29.2–40)

## Discussion

Out of the 104 articles submitted for systematic review and meta-analysis with a sample size of 1,741,613 people, 48 studies were conducted in Asia, 16 studies in Europe, 2 studies in Africa, 32 studies in America, and 6 studies in Oceania. According to the results of the present study, the prevalence of falls in the world's older people was 26.5% (95% CI 29.4.8%). To investigate the effects of potential factors in the heterogeneity of the prevalence of falls in the older people in the world, meta-regression was used for the two factors of the sample size. According to it, with increasing sample size, the prevalence of falls in the older people of the world decreases, which there is a statistically significant difference (*P* < 0.05). Also, with the increase in the research year, the prevalence of falls in the older people of the world decreases, which was also statistically significant (*P* < 0.05). According to the results of subgroup analysis, the highest prevalence of falls in the older people was related to Oceania with 34.4% (95% CI 29.2–40%) and America with 27.9% (95% CI 22.4–34.2%).

Falls are common among the geriatric population; this incident is one of the main causes of disability and death among these people [43, 45]. It is said that those who fall and are not harmed often suffer the negative consequences of that fall. Older people who fall are more likely to fall within a year. These people are also more at risk of falling. This fear of falling can lead to depression and limitation of movement [38].

A study by Boyd, R. et al. showed that 3.5 million people, or about 10 percent of the older people in the USA, have fallen in the past three months. About 1.7 million people were injured, and 875,000 of the injured people went for medical treatment. Based on the results of this study, 12.9 million, or 36%, of the older people in the USA are relatively afraid of falling. According to this study, there is a significant relationship between falling and fear of falling. Among those who recently had a fall, 16% feared a severe or moderate fall; however, only 6% of these people were not afraid or were a little afraid [38].

According to a study by Cevizci, S. et al., those who do not walk at home or out of the house, or walk less, and those who cannot meet their daily needs, have a higher risk of falling than other people. It was also asserted that those who have at least one case of chronic disease, or people with physical and mental impairment, or people with lower quality of life, are at higher risk of falling [40].

The study by Handrigan et al. showed that, according to the dose–response relationship between BMI and prevalence, underweight and obese people were reported to be more common among men. For women, unlike men, obesity was not significantly linked with a higher prevalence of falls [56].

The results of a study carried out by Habibeh Ahmadipour in Kerman, Iran, found that more than a quarter of the older people who referred to the comprehensive health service centers and bases in Kerman during the past 6 months had a history of at least one fall and more than 10 percent also had a history of falling more than once [106]. In astudy by Habibeh Ahmadipour and et al, it was stated that the use of more than four drugs, the use of inappropriate shoes, and the presence of underlying disease were the most common risk factors for health-related in the older people, respectively [106].

With the increase in the elderly population, the need for more care of this population for fractures has increased, because fractures greatly reduce the quality of life of the elderly [107]. Among fractures, pelvic fractures, which occur due to falls in the elderly, are significant, and reports indicate that one-third of patients do not survive more than a year after pelvic fractures [107]. Primary prevention to reduce fractures in the elderly can be done by reducing falls and strengthening bones by eliminating risk factors or by medication [124].

## Conclusion

In conclusion, it is stated that due to the increasing percentage of the world's aging population, the problem of falls, as a common problem with adverse consequences, needs to be seriously considered by policymakers and health care providers to make appropriate plans for interventions and take precautions to reduce falls in the older people. Most of the reasons that lead to falls in the elderly are related to the living environment of the elderly, and by following simple tips and providing assistive equipment to the elderly, the risk of falls in the elderly can be significantly reduced, so appropriate policy to create appropriate living environment for the elderly, such as proper lighting of the house and avoiding total darkening of the house, use of bath chairs and toilets, use of appropriate shoes, not walking after taking sleeping pills, regular eye examinations in the elderly, not carrying heavy equipment, making the phone available, and installing handles in different parts of the house, can help prevent falls in the elderly.

## Data Availability

Datasets are available through the corresponding author upon reasonable request.

## Abbreviations

SID: Scientific Information Database
WoS: Web of Science
STROBE: Strengthening the Reporting of Observational studies in Epidemiology
PRISMA: Preferred Reporting Items for Systematic Reviews and Meta-Analysis
